# Video-assisted thoracoscopic surgery for adult Bochdalek hernia: a case report

**DOI:** 10.1186/s13019-016-0558-0

**Published:** 2016-12-01

**Authors:** Yu-Guang Shen, Na-Na Jiao, Wei Xiong, Quan Tang, Qing-Yong Cai, Gang Xu, Gui-You Liang

**Affiliations:** 1 Department of Thoracic and Cardiovascular Surgery, The First People’s Hospital of Zunyi, Fenghuang Road, Zunyi, Guizhou Province 563000 People’s Republic of China; 2 Department of Nursing, Dalian Road, Affiliated Hospital of Zunyi Medical College, Zunyi, Guizhou Province 563003 People’s Republic of China; 3 Department of Thoracic and Cardiovascular Surgery, Affiliated Hospital of Zunyi Medical College, Dalian Road, Zunyi, Guizhou Province 563003 People’s Republic of China

**Keywords:** Congenital diaphragmatic hernia, Bochdalek hernia, Video-assisted thoracoscopic surgery, Case report

## Abstract

**Background:**

Bochdalek hernia is a type of congenital diaphragmatic hernia that typically presents in childhood, while this diseases is extremely rare in adults.

**Case presentation:**

We review a case of a 63-year-old man with a left-sided Bochdalek hernia who was experiencing occasional pain at the left side of his chest for 8 months. The diagnosis of Bochdalek hernia was made by chest computed tomography. A part of the retroperitoneal adipose tissue was herniated into the left thoracic cavity through the diaphragmatic defect. The hernia was treated via video-assisted thoracoscopic surgery and he made an uneventful recovery.

**Conclusions:**

We report a rare case of a left-sided Bochdalek hernia for which our patient was treated successfully via video-assisted thoracoscopic surgery. Even though rare, this disorder should be recognised, examined and treated appropriately to avoid complications.

## Background

Bochdalek hernias (BH) are a congenital posterolateral diaphragmatic defect, which were first described by Bochdalek in 1848 [1]. Once diagnosed, Bochdalek hernias should be surgically treated during the neonatal period. Therefore, adult cases are rare, with a reported frequency of 0.17 to 6% among all diaphragmatic hernias [2]. Herein we present a case of a 63-year-old man whose Bochdalek hernia was successfully treated by video-assisted thoracoscopic surgery (VATS).

## Case presentation

**Fig. 1:**
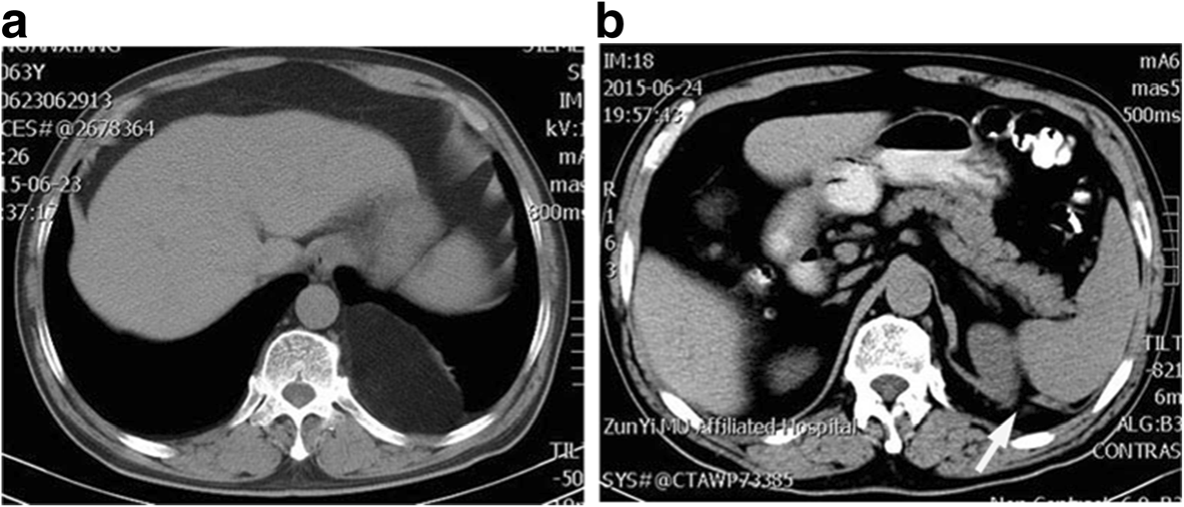
Preoperative chest and abdominal computed tomography (CT) scans. The chest CT shows a posterolateral left-sided mass with fat tissue density (**a**), and the *white arrow* indicates the diaphragmatic defect (**b**)

A 63-year-old man was referred to our hospital because of chest pain. He had experienced occasional pain at the left side of his chest for 8 months. During the past month the pain had become increasingly severe with no hard signs (negative physical examination). The computed tomography (CT) showed a mass in the left thoracic cavity and a left diaphragmatic defect (Fig. 1). According to computed tomography scan and such a location, the diagnosis of a Bochdalek hernia was thus suggested.

The Video-assisted thoracoscopic surgery (VATS) was performed under general anesthesia with single lung ventilation on June 30, 2015. The patient was placed in the right decubitus position, a 15 mm port was made through the 7th intercostal space on the midaxillary line, and then a 30-degree thoracoscope was inserted. Another two ports were next placed at the 5th intercostal space on the anterior axirally line and 7th intercostal space on the scapular line.

**Fig. 2:**
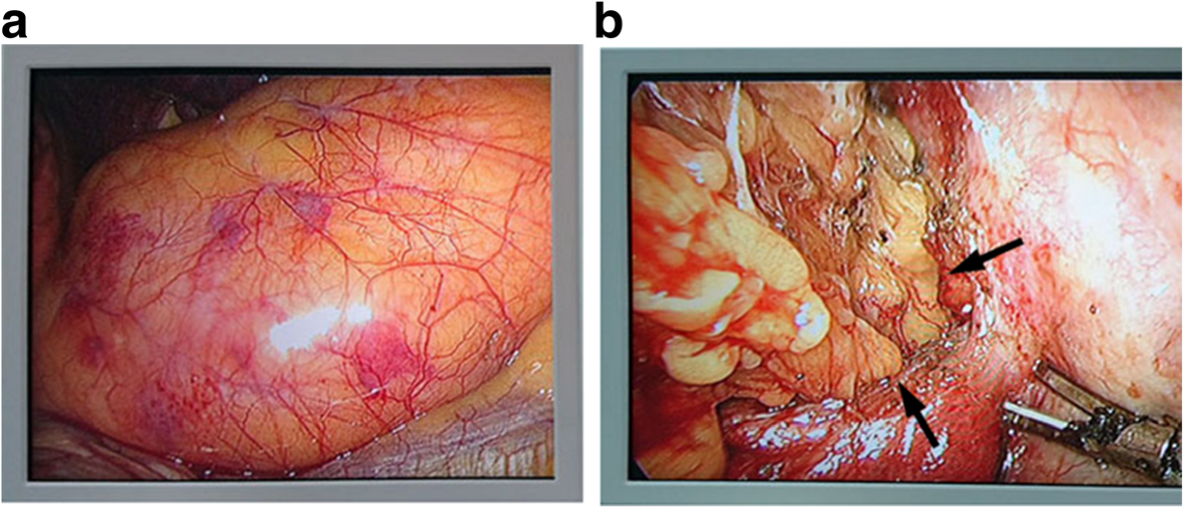
Thoracoscopic view of the Bochdalek hernia. The retroperitoneal adipose tissue was herniated into the thoracic cavity through the diaphragmatic defect (**a**), and the *black arrow* indicates the diaphragmatic defect margins (**b**)

Exploratory thoracoscopy showed that a part of the retroperitoneal adipose tissue was herniated into the left thoracic cavity through the defect (Fig. 2a), 4 cm in length approximately, in the posterior region of the left diaphragm, but no hernia sac was identified. After cutting the parietal pleura enveloping the mass, clear margins of the defect of the diaphragm were revealed (Fig. 2b). The herniated tissue was moved back to the abdominal cavity, and the defect of the diaphragm was then closed with 3-0 polypropylene sutures. The postoperative course was uneventful and the patient was discharged 8 days after the operation without any symptoms. At follow-up, he remains well without any signs of recurrence 6 months after surgery.

## Discussion

BH is a common congenital anomaly in neonatal and postnatal patients and occurs in about one in 2,200 to 12,500 live births, but it is rare in adults [3]. A defective closure of the lumbar and costal muscle groups in the posterolateral diaphragm during the embryonic stage is considered to be the cause of BH. This congenital diaphragmatic hernia is considered to be extremely rare in adults and have a left-sided predominance. Our patient also had left-sided hernia. Complete closure occurs on the right side before it is complete on the left side-a fact that may contribute to the left sided BH being more common than right sided hernias [4]. Most BH are found and repaired in childhood, as many as 5% are first detected in adulthood [5]. BH usually present with severe respiratory distress immediately after birth, which is life-threatening, while most adults present with more chronic symptoms, such as chronic dyspnoea, chest pain and pleural effusion. Recurrent abdominal pain, postprandial fullness and vomiting are the most common abdominal symptoms in adults [6]. Our patient was experiencing the symptoms of recurrent chest pain, while some patients have no symptoms and the disorder is unexpectedly detected on chest X-ray [7].

Diagnosis is usually made on radiological findings. The best radiological investigation in adults is CT scan which has a sensitivity of 78% for left-sided hernia and 50% for the right-sided hernia [8]. Usually, the CT scan show a mass of fat or soft tissue contour of the upper surface of the diaphragm, and a discontinuity of the diaphragm adjacent to the mass. In our case, the diaphragmatic defect could be identified clearly in the computed tomography (CT), and the diagnosis was confirmed. In contrast to Morgagni hernias, BH rarely contains a hernia sac. Only 10.4% patients (18/173) had a hernia sac [4]. Most asymptomatic Bochdalek hernias contain only retroperitoneal adipose tissue, but almost any organ of the peritoneal cavity can herniated through the foramen of Bochdalek [9].

The first successful repair of a BH was performed in 1901 by Aue [10]. The most frequent approach for the Bochdalek hernia is a thoracotomy or transabdominal, and the surgical method is to return the herniated organs to the abdominal cavity and close the diaphragmatic defect [11]. Transthoracic approach is thought to be effective as it allows for direct observation of the herniated viscera or hilium of the hernia or sac, and it is easier to remove the herniated viscera if there is some adherence [12]. With the advancement of modern surgical techniques, less invasive means of repair are available. The VATS is generally considered to be advantageous over standard thoracotomy because it is less invasive, reduced postoperative pain, and allows the surgeon to make more precise incisions. The diaphragmatic defects sometimes do not have sufficient margin for reapproximation directly. Several surgeons reported using artificial material for closing large defect [13, 14]. In our case, the diaphragmatic defect was small (3 cm × 4 cm in size), so we perform primary closure of the diaphragmatic defect by thoracoscopic surgery alone, and we didn’t apply a mesh to reinforce the diaphragm.

## Conclusion

We report a rare case of a left-sided Bochdalek hernia in an adult who was treated via VATS. People with a Bochdalek hernia may not have any symptoms and the disorder may be detected unexpectedly, or the symptoms and expressions may vary from mild to serious complications. Even though rare, this disorder should be recognised, examined and treated appropriately to avoid complications.

## Abbreviations

BH: Bochdalek hernia
CT: Computed tomography
VATS: Video-assisted thoracoscopic surgery
